# Bilingualism and Naming in MCI and Alzheimer's Disease: Insights from Catalan‐Spanish Speakers

**DOI:** 10.1002/alz70857_106102

**Published:** 2025-12-25

**Authors:** Marco Calabria, María Sainz‐Pardo, Mireia Hernández, Anna Suades, Montserrat Juncadella, Jordi Ortiz‐Gil, Lidia Ugas, Isabel Sala, Alberto Lleó

**Affiliations:** ^1^ Faculty of Health Sciences, Universitat Oberta de Catalunya, Barcelona, Barcelona, Spain; ^2^ Universitat Oberta de Catalunya, Barcelona, Barcelona, Spain; ^3^ University of Barcelona, Barcelona, Spain; ^4^ ENTIA, Fundació de Neurorehabilitació i Recerca Cognitiva, Barcelona, Barcelona, Spain; ^5^ ENTIA, Fundació de Neurorehabilitació i Recerca Cognitiva, Barcelona, Spain; ^6^ Universitat de Vic‐Universitat Central de Catalunya, Vic, Spain; ^7^ FIDMAG Germanes Hospitalàries Research Foundation, Barcelona, Spain; ^8^ Hospital Benito Menni, CASM, Sant Boi de Llobregat, Spain; ^9^ Sant Pau Memory Unit, Hospital de la Santa Creu i Sant Pau ‐ Biomedical Research Institute Sant Pau ‐ Universitat Autònoma de Barcelona, Barcelona, Cataluña, Spain; ^10^ Sant Pau Memory Unit, Department of Neurology, Hospital de la Santa Creu i Sant Pau, Institut d'Investigació Biomèdica Sant Pau (IIB SANT PAU), Facultad de Medicina ‐ Universitat Autònoma de Barcelona, Barcelona, Spain; ^11^ CIBERNED, Network Center for Biomedical Research in Neurodegenerative Diseases, National Institute of Health Carlos III, Madrid, Spain

## Abstract

**Background:**

Evidence consistently indicates a disadvantage in bilinguals' speech production compared to monolinguals in healthy individuals, particularly for low‐frequency words. However, research on this phenomenon in clinical populations, such as those with Mild Cognitive Impairment (MCI) and Alzheimer's Disease (AD), remains scarce. This study investigates the impact of bilingualism on naming within the Catalan‐Spanish language pair, two typologically similar Romance languages, while also comparing active and passive bilinguals.

**Method:**

A total of 124 patients with MCI, 66 patients with AD, and 58 healthy older adults participated in the study. Participants were classified as either Catalan‐Spanish early and highly proficient bilinguals (active bilinguals) or Spanish‐dominant speakers with low proficiency in Catalan (passive bilinguals). A picture‐naming task in the participants’ dominant language was used, with stimuli selected based on frequency (high vs. low) and phonological overlap (cognates vs. non‐cognates). Naming performance and accuracy were analyzed using linear mixed models and logistic regressions, respectively.

**Result:**

For naming latency, the frequency effect (high vs. low) increased significantly across groups. There was a 521 ms increase (95% CI: [423, 691], z = 10.46, p < .001) from healthy older adults to MCI participants and a 580 ms increase (95% CI: [481, 678], z = 11.06, p < .001) from MCI participants to AD patients. For accuracy, the frequency effect also increased significantly between groups, with a 6.7% decrease in accuracy (95% CI: [5.3, 8.2], z = 9.97, p < .001) from older adults to MCI participants and a further 14.0% (95% CI: [11.9, 16.0], z = 13.58, p < .001) from MCI participants to AD patients. However, the frequency effect was not influenced by the type of bilingualism.

**Conclusion:**

The results from Catalan‐Spanish bilinguals with MCI and AD do not align with the weaker link hypothesis or competitive models of bilingual language production. A possible explanation lies in the language context: passive bilinguals, who are regularly exposed to a second language, may differ fundamentally from monolinguals examined in prior studies. This continuous exposure to a second language could influence cognitive and linguistic processing in ways not accounted for in monolingual comparisons.

